# Transforming Motor Imagery Analysis: A Novel EEG Classification Framework Using AtSiftNet Method

**DOI:** 10.3390/s24196466

**Published:** 2024-10-07

**Authors:** Haiqin Xu, Waseem Haider, Muhammad Zulkifal Aziz, Youchao Sun, Xiaojun Yu

**Affiliations:** 1 College of Civil Aviation, Nanjing University of Aeronautics and Astronautics, Nanjing 211106, China; chuyi809@nuaa.edu.cn (H.X.); sunyc@nuaa.edu.cn (Y.S.); 2 School of Automation, Northwestern Polytechnical University, Xi’an 710072, China; waseemhaider@mail.nwpu.edu.cn (W.H.); zulkifalaziz@mail.nwpu.edu.cn (M.Z.A.)

**Keywords:** brain–computer interface (BCI), attention sift network (AtSiftNet), motor imagery (MI), independent component analysis (ICA), principal component analysis (PCA)

## Abstract

This paper presents an innovative approach for the Feature Extraction method using Self-Attention, incorporating various Feature Selection techniques known as the AtSiftNet method to enhance the classification performance of motor imaginary activities using electrophotography (EEG) signals. Initially, the EEG signals were sorted and then denoised using multiscale principal component analysis to obtain clean EEG signals. However, we also conducted a non-denoised experiment. Subsequently, the clean EEG signals underwent the Self-Attention feature extraction method to compute the features of each trial (i.e., 350×18). The best 1 or 15 features were then extracted through eight different feature selection techniques. Finally, five different machine learning and neural network classification models were employed to calculate the accuracy, sensitivity, and specificity of this approach. The BCI competition III dataset IV-a was utilized for all experiments, encompassing the datasets of five volunteers who participated in the competition. The experiment findings reveal that the average accuracy of classification is highest among ReliefF (i.e., 99.946%), Mutual Information (i.e., 98.902%), Independent Component Analysis (i.e., 99.62%), and Principal Component Analysis (i.e., 98.884%) for both 1 and 15 best-selected features from each trial. These accuracies were obtained for motor imagery using a Support Vector Machine (SVM) as a classifier. In addition, five-fold validation was performed in this paper to assess the fair performance estimation and robustness of the model. The average accuracy obtained through five-fold validation is 99.89%. The experiments’ findings indicate that the suggested framework provides a resilient biomarker with minimal computational complexity, making it a suitable choice for advancing Motor Imagery Brain–Computer Interfaces (BCI).

## 1. Introduction

In recent years, we have seen incredible progress in the field of big data applications and artificial intelligence, which become the main reason for the creation of ground-breaking technologies that link computers with the human brain while the mind-control wheelchair, prosthetic arms, home automation system, etc., are the innovations of BCI in healthcare sectors [1,2,3]. The low potential signals generated in the brain during any neural activity have a significant amount of information, which we can decode in the field of BCI and convert that data into meaningful information.

In the case of Motor imagery, it only consists of those signal that involves activation of the neural system when a person without any body movement or physical activity imagines performing a task. When a person performs a task without moving his/her body physically then it is known as a cognitive process, and MI is defined as a cognitive process because it does not have any physical movement [4,5]. For example, in our case, the MI task in BCI competition III dataset IV-a is “right-hand movement” and “right-foot movement”. Given the characteristics required for motor imagery applications, which involve a low-cost, noninvasive, and high temporal resolution signal retrieval method [3,6], electroencephalography (EEG) stands out as the optimal selection due to its inherent possession of these characteristics. Once the signal is obtained from the subject performing the MI task, another challenge is to accurately interpret it into meaningful classification [7].

Motor imagination is a very important concept in the Brain–Computer interface (BCI), it is a rapidly advancing topic of research that aims to provide direct communication between the brain and the real world. In the case of the human brain, it is a sophisticated organ with extensive networks that manage a variety of tasks, including motor control. If a person imagines herself/himself conducting a motor activity, such as moving their hand or foot, the brain areas linked with real motor neuron execution are active. These imagined actions create brain signals that BCI devices can collect and identify. One of the primary benefits of motor imaging in BCI is that it does not need real body motion, making it especially beneficial for those with motor limitations. BCI systems that use motor imagery have shown potential in offering communication and control actions for patients suffering from spinal cord injuries, amyotrophic lateral sclerosis (ALS), or paralysis.

Electroencephalography (EEG), a non-invasive technique that captures the electrical responses of the brain using electrodes applied to the scalp is commonly used to integrate motor imagery in BCI. Certain frequency bands, such as beta and mu rhythms, show amplitude and synchronization alterations during motor imagery. Then, these modifications are employed as features to interpret the user’s intents. Numerous facets of motor imagery-based BCI research have been investigated, such as machine learning algorithms for classification, feature extraction approaches, and signal processing strategies. Technological developments in signal processing have improved the ability to extract appropriate data from EEG signals, and machine learning techniques are essential for converting these signals into instructions that devices can understand. Research has shown that motor imagery-based BCIs are feasible for a range of uses. For example, people with locked-in syndrome or paralysis have effectively employed motor imagery to operate robotic arms, computer cursors, and even spell words using equipment. The quality of life for those with significant motor disabilities might be greatly enhanced by using motor imagery for BCI applications. The variance of individual brain patterns, the requirement for intensive user training, and the sensitivity of EEG signals to artifacts as well as noise are challenges faced by motor imagery-based BCIs. Through advancements in adaptive algorithms, neurofeedback training paradigms, and signal processing, researchers are actively attempting to overcome these problems.

In this study, we begin with the sorting and denoising of Motor Imagery EEG data, which are crucial for enhancing the quality of the signals and reducing noise that can interfere with accurate classification. Following this preprocessing step, we extract features using self-attention deep learning methods, which allow us to identify and prioritize the most relevant aspects of the EEG signals.

After feature extraction, we employ various feature selection techniques, including Mutual Information and Recursive Feature Elimination (RFE), to further refine the dataset. These methods help in identifying the most informative features, thereby enhancing the classification performance while reducing computational complexity.

Subsequently, we classify the processed data using both traditional machine learning methods and the latest deep learning techniques. In the machine learning domain, we utilize classifiers such as Support Vector Machines (SVM), k-Nearest Neighbors (k-NN), and Linear Discriminant Analysis (LDA). For deep learning, we implement advanced models including Recurrent Neural Networks (RNN), Gated Recurrent Units (GRU), WebNet, CatBoost, and ResNet. By integrating these approaches, we aim to provide a comprehensive evaluation of the effectiveness of each model in classifying Motor Imagery EEG data, ultimately contributing to advancements in Brain–Computer Interface (BCI) systems.

To the best of our knowledge, this is the first time that we are using a novel Attention Sift Network (AtSiftNet) technique to enhance the classification accuracy of motor imagery tasks in the field of Brain–Computer Interface. The conventional methods such as convolutional neural networks (CNNs) and recurrent neural networks (RNNs) present several limitations in this context [8,9]. CNNs, while effective at capturing spatial patterns, often require large datasets and extensive training time, making them computationally expensive and time-consuming. Moreover, they can struggle with the inherent variability and noise in EEG signals. RNNs, on the other hand, are designed to process sequential data but often face challenges in retaining long-term dependencies, limiting their ability to accurately process the temporal dynamics of motor imagery data. AtSiftNet addresses these limitations by introducing an architecture that leverages self-attention mechanisms to focus on the most relevant features of the signal while reducing overall computational complexity. This approach not only improves the accuracy of motor imagery classification but also accelerates the processing time, making it more suitable for real-time applications. By bridging this gap, AtSiftNet offers a more efficient and effective solution compared to traditional CNNs and RNNs in motor imagery-based BCI systems [10]. The fundamental premise of our approach lies in leveraging the unique capabilities of self-attention for MI data analysis. Self-attention mechanisms have demonstrated exceptional efficacy in capturing long-range dependencies within sequential data; by focusing on capturing relevant spatial and temporal relationships within the MI data, we aim to unlock richer and more discriminative features that can significantly improve classification accuracy. The feature selection phase of our methodology is a critical step in distilling the most informative elements from the extracted features. We employed a diverse set of eight feature selection methods to systematically identify and prioritize features that contribute most significantly to the classification task. These methods encompass Mutual Information Feature Selection (MIFs) [11], Recursive Feature Elimination (RFE), Linear Regression (LR), Neighborhood Component Analysis (NCA) [12], Relief Feature Selection (ReliefF) [13], Independent Component Analysis (ICA) [14,15], Principal Component Analysis (PCA) [16], and Correlation Feature Selection (Cor) [17].

The inclusion of these diverse feature selection techniques ensures a comprehensive exploration of the feature space, allowing us to capture both linear and non-linear relationships within the MI data. Moving beyond feature extraction and selection, our paper introduces a robust ensemble of five classification methods. These methods, namely Support Vector Machine (SVM) [18], Linear Discriminant Analysis (LDA) [19], k-Nearest Neighbors (KNN) [20], Random Forest (RF) [21], and XGBoost Classification [22,23], represent a well-rounded suite of algorithms known for their efficacy in handling complex classification tasks.

The integration of multiple classifiers enables a comparative analysis of their performance, shedding light on the strengths and weaknesses of each method in the specific context of MI data classification. By combining state-of-the-art feature extraction, diverse feature selection strategies, and a suite of advanced classifiers, our research aims to contribute to the field of BCI systems. The interdisciplinary nature of our approach, merging techniques from signal processing, machine learning, and neuroscience, reflects a holistic effort to enhance the interpretability and accuracy of MI data analysis. Through this exploration, we aspire to pave the way for advancements in BCI systems, opening new possibilities for real-world applications in healthcare, rehabilitation, and human-computer interaction.

## 2. Materials and Methods

### 2.1. Analyzed Data

The MI data that we used in this study are the publicly available dataset BCI competition three dataset IVa. This dataset consisted of five healthy subjects as a volunteer, named “aa, al, av, aw, and ay”, and their main task was to imagine the right hand (Class 1) and right foot (Class 2) movement, without any physical movement. The dataset was collected by using 118 EEG electrodes as per the 10/20 international system, and the total number of tails that were taken in this dataset was 280, with 140 trials in each class. For 3.5 s each subject is shown his/her visual sign and the sampling rate of the dataset is 100 Hz [5].

### 2.2. Software and Hardware Environment

The experiments were conducted using a combination of MATLAB R2022b and Spyder within the Anaconda3 distribution. MATLAB R2022b was employed for data sorting and denoising due to its robust environment. Python coding was performed in Spyder, with the choice of Python driven by the accessibility of powerful libraries such as torch and torch.nn, which were utilized to implement self-attention mechanisms in the experiments. The system used for the experiments was equipped with an Intel(R) Core(TM) i5-6400 CPU operating at 2.70 GHz, supported by 8.00 GB of RAM (7.88 GB usable). The machine also ran on a 64-bit architecture with an x64-based processor and included Intel(R) HD Graphics 530, providing 4 GB of GPU memory. This configuration ensures reliable performance for the processing tasks, and the details provided are intended to enhance the reproducibility of the experiments.

### 2.3. Methods

**Figure 1:**
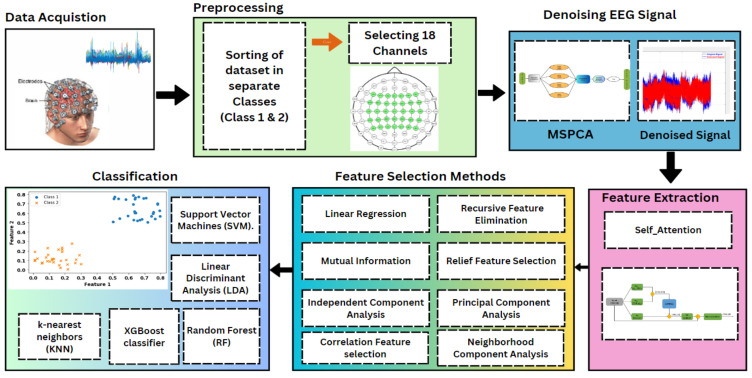
Proposed Framework for novel AtSiftNet method.

In this paper, we present our novel AtSiftNet method in which we used a Self-attention Feature extraction method with eight feature selection and five classification methods, to classify motor imagery EEG signals. To the best of our knowledge, for the first time, this study introduced a novel AtSiftNet technique in the field of BCI for the efficient classification of MI tasks of EEG signals. The proposed framework of this study is clearly depicted in Figure 1. In this study, the proposed approach first sorts the data according to the description of the dataset, in which class 1 is the right-hand movement, and class 2 is the right foot movement separated on the base of dataset description, and finally, in the preprocessing step only 18 electrodes were selected out of 118 electrodes [24], and then the selected channels were denoised by using MSPCA technique. Later on, self-attention feature extraction and eight feature selection techniques are used to extract the features from each trial, and then selected features are used as the inputs to several machine learning and neural network classifiers.

#### 2.3.1. Step 1: Denoising by Using MSPCA

In this study, we opted for Multi-Scale Principal Component Analysis (MSPCA) as the primary denoising technique for EEG signals in motor imagery-based Brain–Computer Interfaces (BCIs). Although alternative methods such as empirical mode decomposition (EMD) and wavelet thresholding have been widely used in similar applications, MSPCA offers distinct advantages that make it particularly well-suited for this task.

MSPCA efficiently combines both time and frequency domain analysis, which is critical for handling the non-stationary nature of EEG signals. By decomposing the signal into multiple scales and applying principal component analysis (PCA) at each scale, MSPCA is able to retain essential features across different resolutions while effectively reducing noise. This multi-scale approach provides a more robust framework for signal denoising in comparison to single-scale methods like wavelet thresholding, which can be sensitive to the choice of wavelet basis and thresholding techniques, potentially resulting in the loss of important signal components.

**Figure 2:**
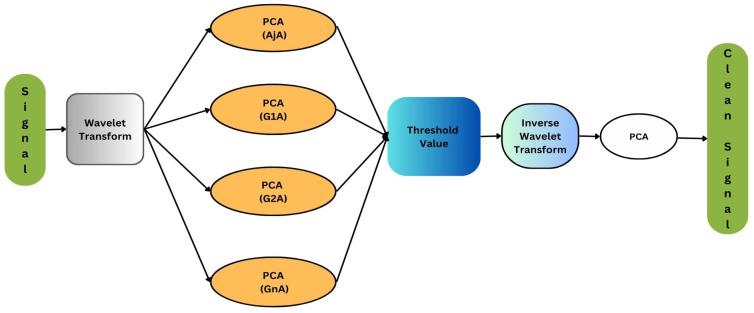
Block Diagram of (MSPCA).

A weak and fragile EEG signal was obtained when we recorded that signal from a participant’s scalp, and these weak signals were also prone to different types of noise content, for example, electromyography (EMG), blink signal noise, power line noise, etc. The mathematical model of this noise can be represented as

$$P=P_{o}+P_{x}$$

where $P_{o}$ is the clean signal that we want, while the $P_{x}$ is the noise artifact signal that overlaps with the original signal during data collection. Principal component analysis (PCA) is the traditional method that can extract the clean EEG signal $P_{o}$ without affecting it, this model’s main objective is to remove the noise artifacts without affecting the $P_{o}$ contents. PCA determined the correlated data points and found the linear relationship between classes. As the EEG signals are non-linear and non-stationary it needs to convert those signals into time-frequency wavelets, and this requires we can be achieved by using wavelet transform, because of its effectiveness. With the help of PCA and wavelet transform we can build a denoising algorithm as shown in Figure 2.

In this algorithm for denoising the matrix *A* which contains the EEG signal, first decompose the signal into *N* levels by using wavelet transform (W.T), then the *N* level is passed through the PCA to calculate detailed matrix AjA and approximation matrix GjA, then apply the inverse wavelet transform on the resultant principal components.

**Figure 3:**
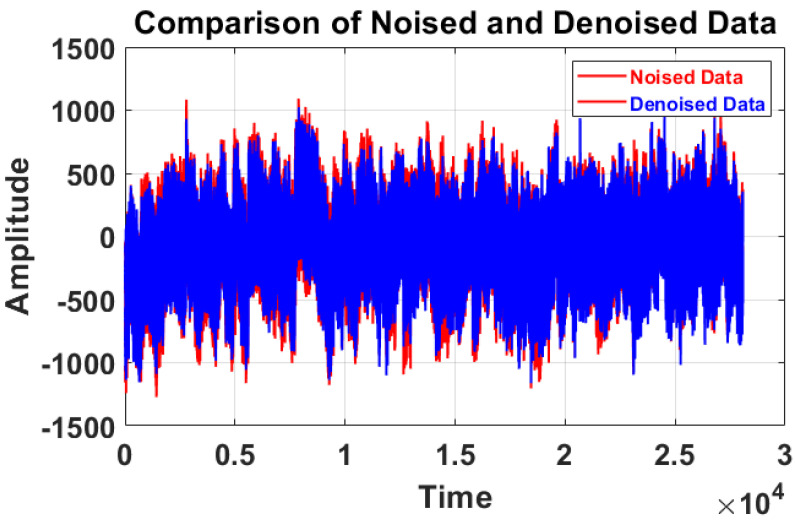
A comparison graph between original EEG signal and Denoised signal.

Finally, the resultant principal components obtained in the previous step undergo PCA again to extract the complete denoised EEG data/signal. In this study, we used the Sym4 wavelet. In the previous framework, only specific main components with eigenvalues higher than the mean of collective eigenvalues were chosen, following the Kaiser law. After multiple trials, five wavelet levels were selected. The detail and approximation signals were generated by the Sym4 wavelet, chosen empirically [25]. In Figure 3, we can clearly observe that after using MSPCA, the signal is clean, and its amplitude is reduced due to the denoising process.

#### 2.3.2. Step 2: Channel Selection

The original dataset, stored in the “cnt matrix”, was gathered using 118 electrodes, constituting a substantial amount of data. Processing such a large dataset resulted in increased computational time, heightened system complexity, and information redundancy. Therefore, in this paper, we opted to select only 18 channels from the original 118, specifically C5, C3, C1, C2, C4, C6, CP5, CP3, CP1, CP2, CP4, CP6, P5, P3, P1, P2, P4, and P6 [24]. These electrodes are strategically positioned around the motor cortex region following the 10/20 international system. The reason behind this selection is rooted in the fact that motor imagery (MI) signals predominantly originate in the brain’s motor cortex [25]. Consequently, these 18 electrodes contain enough information pertinent to the specific MI task, facilitating subsequent processes such as feature extraction, feature selection, and classification.

#### 2.3.3. Step 3: Self-Attention Feature Extraction

In terms of feature extraction, self-attention can be viewed as a weighted combination of input patterns. The relevance of each data to others determines the weight, enabling the model to prioritize important data over irrelevant ones. Additionally, this model effectively captures hierarchical and long-range dependencies. The self-attention model involves three basic vectors: the key ($\omega_{k}$), query ($\omega_{q}$), and value ($\omega_{v}$). These vectors, i.e., $\omega_{k}$, $\omega_{q}$, and $\omega_{v}$, are learned during the training process.

During the calculation of attention scores, the model performs the dot product of the query vector of one signal with the key vector of another signal. To prevent excessive numbers that could lead to signal instability, the scores are then scaled by the square root of the dimension of the key vectors. The SoftMax function is applied to the attention scores to obtain weights, indicating the significance or value of each data with respect to the current term ([26]).

In this study, we employed self-attention to extract meaningful features from each trial of our Motor Imagery (MI) EEG dataset. Each trial consists of 350 time samples, representing a sequence of brain activity recorded over time. These samples are collected from 18 different channels, each corresponding to specific regions of the cortex, where electrical signals are captured to monitor brain activity during motor imagery tasks. The choice of these 18 channels is based on their relevance to motor control and imagery, which ensures that the data capture the key neural patterns associated with motor intentions.

Thus, for each trial, the input data forms a matrix of size (350, 18), where 350 represents the number of time steps in the trial, and 18 represents the different EEG channels. Each row in this matrix corresponds to a specific time point, and each column represents the signal recorded from a particular brain region at that time. This multi-dimensional input provides rich temporal and spatial information about the brain’s activity during motor imagery.

The self-attention mechanism is applied to this matrix to automatically learn and extract the most relevant features from the EEG signals. By calculating attention scores between different time points and channels, the model identifies which parts of the signal should be prioritized based on their importance in relation to others. For example, the attention mechanism can highlight key moments in the EEG data, such as bursts of neural activity, while downplaying noise or less relevant information. This is crucial, as EEG signals are often highly noisy and variable, making manual feature extraction difficult and prone to error.

Moreover, the self-attention mechanism not only focuses on local patterns within the signal but also captures long-range dependencies. This is particularly important in motor imagery tasks, where both immediate brain responses and more sustained patterns of neural activity might be relevant for distinguishing between different motor tasks. By assigning attention weights to different parts of the trial, the model effectively balances the importance of short-term and long-term signal patterns.

Each trial’s input matrix of (350, 18) allows the self-attention model to process the raw EEG data in a structured way, extracting the most important features by leveraging the relationships between time samples and channels. This process enhances the model’s ability to interpret complex EEG data, leading to better downstream classification of motor imagery tasks.

**Figure 4:**
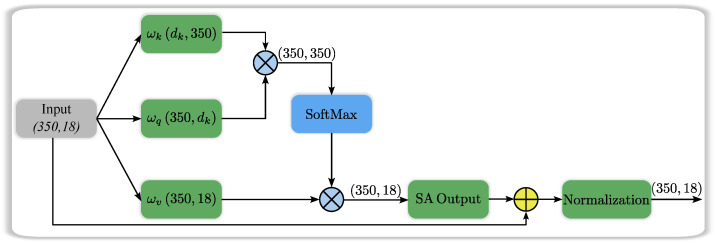
Architecture of Self-attention feature extraction.

The steps involved in this technique for each trial are described as follows:

1. Linear transformation: By employing linear layers (nn.Linear), we transform the input tensor *X*, which has dimensions (350,18), into three distinct spaces: query, key, and value. Through these learned transformations, input characteristics are projected into spaces conducive to obtaining meaningful representations. Specifically, the dimensions for queries become (350, $d_{k}$), keys adopt the dimensions of ($d_{k}$, 350), and values maintain the dimensions of (350,18). This process is performed for a single trial of our MI signal. Here, the three vectors Query *Q*, Key *K*, and Value *V* are the transformed input sequence “*X*”:
  $$Q_{i}=XW_{Q}$$
  $$K_{i}=XW_{K}$$
  $$V_{i}=XW_{V}$$
  where $W_{Q}$, $W_{K}$, and $W_{V}$ are the learnable weight matrices.
2. Dot-product attention: Dot-product attention involves calculating the dot product of the query tensors $\left(350,d_{k}\right)$ and the key tensors $\left(d_{k},350\right)$. This computational process helps identify correlations and similarities among components in the input sequence of the MI signal. If the value of the dot product is larger, it indicates greater importance of the data. Following the dot product, the dimension becomes (350,350) for each trial, as illustrated in Figure 4. Mathematically,
  $$D\left(Q,K\right)=Q\;\cdot \;K^{T}$$
3. Scaling: In this step, the dot-product scores were scaled with the square root of the key vector dimensionality $d_{k}$. This scaling helps prevent the numerical instability that may arise in our motor imaginary (MI) signal. The scaling stage in the self-attention technique specifically addresses the potential numerical instability during the calculation of the dot product. The dot product, a mathematical operation involving the multiplication and addition of corresponding elements in two vectors, is computed for the query $\left(350,d_{k}\right)$ and key $\left(d_{k},350\right)$ tensors within the self-attention framework. In summary, the scaling employed in the self-attention model serves as a precautionary measure to maintain numerical stability in our proposed model during the computation of the dot product.
  $$Scaling\left(Q,K\right)=\frac{Q\;\cdot \;K^{T}}{\sqrt{d_{k}}}$$
4. SoftMax: After scaling, the SoftMax function takes the vector of real numbers and converts them into a probability distribution. In the case of self-attention feature extraction, each row of the tensor along its final dimension receives a separate application of the SoftMax function. This final dimension, in the case of self-attention, matches the components in the input sequence. The raw scores are converted into a probability distribution using the SoftMax method. A larger probability corresponds to MI data with larger-scaled dot-product scores, suggesting a greater level of significance or attention. Hence, the SoftMax operation converts the MI data into attention weights to normalize the scores, and the importance and attention assigned to each MI data from the input sequence with the other data are represented by these weights obtained after SoftMax. The attention weights are computed as follows:
  Attention_Weight=softmax(Scaling(Q,K))
5. Weighted multiplication of Values: The attention weights obtained in the previous SoftMax step (350, 350) for one trial of the MI signal are now multiplied by the weighted sum of the value of key vector ⋯. This step combines different parts of integrated data from various segments of the MI input sequence, assigning greater weight to input segments considered more relevant or significant based on the attention weights.
  Output=Attention_Weight.V
6. Sum with Input and Normalization: After multiplying the SoftMax and weighted values, the tensor flow matrix of (350, 18) is formed, as illustrated in Figure 4. Subsequently, in the next step, we add this value to the input of the denoised EEG signal, which shares the same dimensions of (350, 18). Following the addition of the SoftMax value to the input MI signal, we apply the min-max normalization technique. This technique scales every MI data point to a specific range of [0,1]. It is important to note that this normalization technique is commonly employed to standardize data, facilitating the comparison and manipulation of values with initially disparate scales.

#### 2.3.4. Step 4: Feature Selection

After extracting features through self-attention, the next step involves selecting the optimal feature from the MI signal during the feature selection process. It is worth noting that, following feature extraction, the samples in each trial are of dimension (350, 18). In this study, eight feature selection methods were employed, each choosing either a single or fifteen samples from each trial. Consequently, after feature selection, the dimensions of each MI signal trial become X (1, 18) when only the best feature is selected, and X (15, 18) when the top fifteen best feature signals are chosen. The eight feature selection methods utilized in this paper are outlined below:

1. Linear Regression (LR): In Linear Regression, the relationship between a dependent variable and one or more independent variables by fitting a linear equation to observed data. In our case, which is the case of feature selection, it evaluates the contribution of each feature to the overall prediction and selects features based on their regression coefficients.
2. Relief Feature Selection (ReliefF): ReliefF is a machine learning algorithm designed for feature selection in classification problems. It evaluates the importance of features by considering the difference in feature values between instances of the same and different classes. Features that contribute remarkably to class discrimination are retained.
3. Mutual Information (MIg): Mutual Information is a measure of the dependence between two random variables. In the case of feature selection, mutual information (MIg) estimates the information shared between each feature with its target variable, providing an assessment of the relevance of each feature to the classification task of MI data.
4. Correlation Feature Selection (COR): Correlation Feature Selection quantifies the correlation between each feature and the target variable. It selects features with high correlation, and designation of a strong relationship with the target. This method is specifically effective when dealing with linear relationships of MI data.
5. Recursive Feature Elimination (RFE): RFE is a backward elimination technique where features are recursively eliminated on the base of their importance. It involves training a model, ranking features of each iteration by their importance, and eliminating the least important features in each iteration until the desired results which is the number of features were obtained.
6. Principal Component Analysis (PCA): The principal component analysis ‘PCA’ is a dimensionality reduction method that alters the original features of our MI data into a new set of uncorrelated variables, which are called principal components. In feature selection, the top principal components have the main information and it will be retained while others are discarded.
7. Neighborhood Component Analysis (NCA): Neighborhood component analysis ‘NCA’ is a technique that pursues to find a linear transformation of the data that enhances the classification accuracy. It focuses on maintaining the neighborhood relationships between data points of each trial and emphasizes those features that contribute to effective discrimination.
8. Independent Component Analysis (ICA): Independent component analysis is a method that segregates a multivariate signal into additive, independent components. In the case of feature selection, ICA is recognized as those components that seize unique and non-redundant information, making it particularly beneficial for extracting independent features.

#### 2.3.5. Step 5: Classification

In this paper, we conducted a comprehensive assessment of our proposed method to ensure a fair evaluation of its performance of MI data. To accomplish this milestone, we used a five-fold cross-validation technique and utilized five well-defined classification methods as well. The matrix that we obtained after feature selection for each subject was randomly divided into five subsets or folds, and the cross-validation procedure was then repeated five times. During each iteration, a different fold was delegated as the test set, while the remaining folds were used as the training set. This careful approach not only enhances the solidity of our evaluation but also enables the detection of potentially noisy data in the method that we used in our paper.

To further extend and diversify our evaluation, we used the following five classification methods:

1. Support Vector Machine (SVM): Support Vector Machine is an influential and powerful supervised learning algorithm worthy for both classification and regression tasks of MI data. The SVM classification algorithm works by locating the hyperplane that best separates the data into different classes, and then maximizing the margin between classes (i.e., class 1 and class 2). SVM is particularly effective in high-dimensional spaces.
2. Linear Discriminant Analysis (LDA): Linear Discriminant Analysis is a dimensionality reduction and classification technique. It aims to find the linear combinations of features that best separate multiple classes. LDA is particularly advantageous when the classes are well-separated and the assumptions of normality are met.
3. k-Nearest Neighbors (KNN): k-Nearest Neighbors is a non-parametric and instance-based learning algorithm used for classification and regression. It classifies a data point by considering the class labels of its k nearest neighbors. KNN is simple yet robust, and its performance can be influenced by the choice of the distance metric.
4. Random Forest (RF): Random Forest is an ensemble learning method that constructs a multitude of decision trees during training and outputs the mode of the classes for classification problems. It excels in handling complex relationships in data, and its ensemble nature enhances robustness and reduces overfitting.
5. XGBoost Classification: XGBoost is a popular gradient-boosting algorithm known for its efficiency and accuracy. It sequentially builds a series of weak learners and combines them to create a strong learner. XGBoost is versatile, handles missing data well, and is less prone to overfitting.

By incorporating these diverse classification methods in conjunction with the five-fold cross-validation, we aimed to thoroughly assess the robustness and generalizability of our proposed method across different algorithmic frameworks. This strategy enhances the comprehensiveness of our study and contributes to a more nuanced understanding of the method’s performance.

To evaluate the experimental results following the five-fold validation and the application of five different classification methods, we assessed various statistical parameters such as accuracy, sensitivity, and specificity. The formulas for these parameters are provided below:

$$Accuracy=\frac{TP+TN}{TP+TN+FP+FN}$$

$$Sensitivity=\frac{TP}{TP+FN}$$

$$Specificity=\frac{TN}{TN+FP}$$

The terms used in this paper, “true positives” (*TP*) and “true negatives” (*TN*), represent the number of correctly classified occasions for class 1 and class 2, respectively. On the other hand, false positives (*FP*) and false negatives (*FN*) indicate the number of incorrectly classified occasions for class 1 and class 2 [5].

## 3. Results and Discussion

### 3.1. Analysis of Five Classifiers

**Figure 5:**
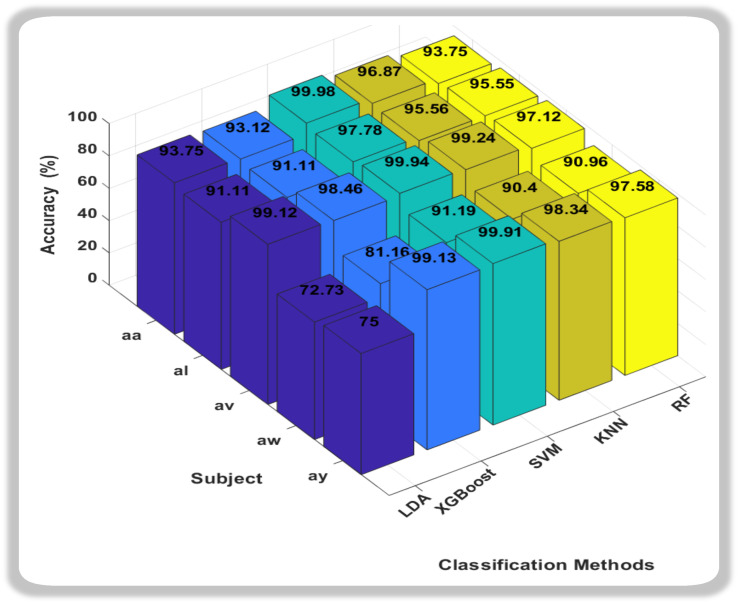
Classification accuracy (%) of all subjects and machine learning classifier.

In this study, we assessed the efficacy and accuracy of five classification algorithms—Linear Discriminant Analysis (LDA), XGBoost, Support Vector Machine (SVM), k-nearest neighbors (KNN), and Random Forest (RF)—for MI classification within the framework of five subjects (aa, al, av, aw, ay) within our dataset. The accuracy results revealed distinct patterns among the classifiers. Linear Discriminant Analysis (LDA) showed consistent accuracy, while XGBoost demonstrated competitive performance. The detailed 3D bar graph of the Accuracy and classification method is shown in Figure 5.

**Figure 6:**
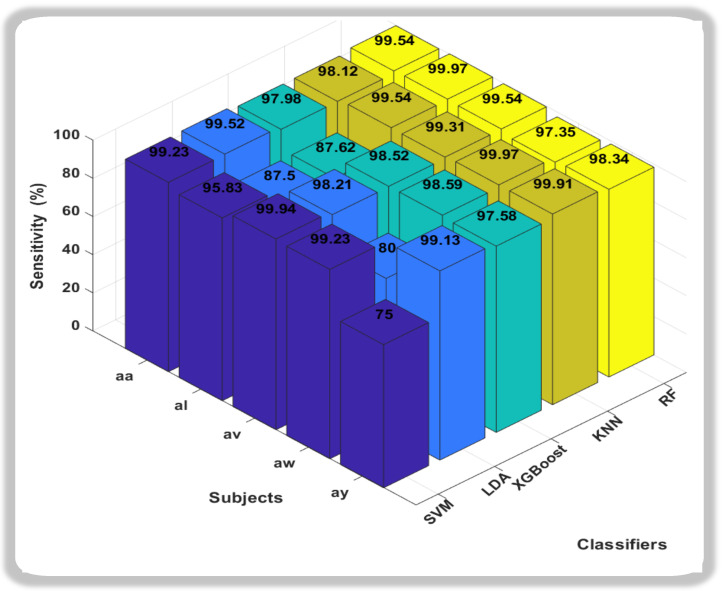
3D bar chart of Sensitivity by using different classifiers.

**Figure 7:**
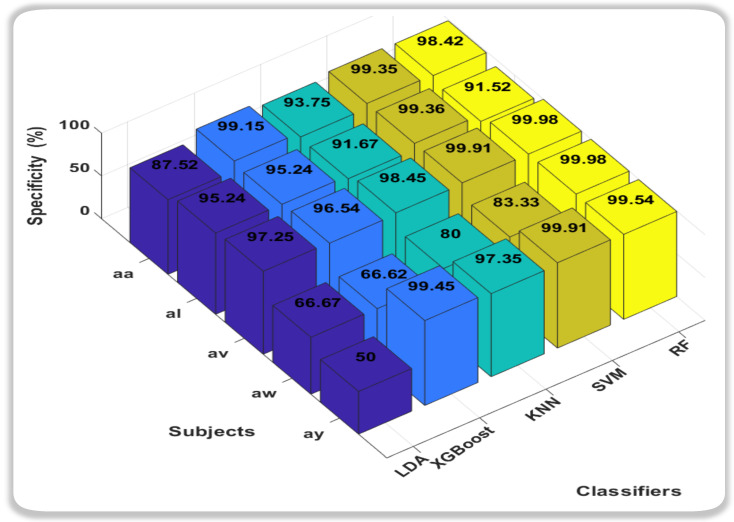
Specificity of all subjects of MI task with all classifiers.

In this novel method, the accuracies we obtain from all classification methods were outstanding, but it was the Support Vector Machine (SVM) that consistently outperformed other classifiers, showcasing remarkably high accuracy across all subjects. Notably, SVM achieved near-perfect accuracy for subjects aa, av, and ay, indicating its robustness in discerning subtle patterns within diverse datasets. Random Forest (RF) also exhibited strong performance, particularly excelling in subject av. While not consistently surpassing SVM, RF demonstrated competitive accuracy across subjects, highlighting its adaptability to different datasets. In conclusion, both SVM and RF emerge as strong candidates for the best classifiers, with SVM demonstrating exceptional accuracy across subjects, and RF showcasing robust performance, especially in subject av. The choice between SVM and RF may hinge on specific considerations such as interpretability, computational efficiency, or the need for a well-generalized model. Further analysis and validation could provide additional insights into the nuanced performance of these classifiers for motor imagery classification tasks. We also calculated the Sensitivity and Specificity of each subject with respect to classifiers as shown in Figure 6 and Figure 7.

In the realm of sensitivity and specificity analysis for motor imagery classification in this study, two classifiers, k-nearest neighbors (KNN) and Random Forest (RF), consistently demonstrate superior performance compared to their counterparts. When focusing on sensitivity, which measures the ability of a classifier to correctly identify positive instances, both KNN and RF emerge as robust choices. Their capacity to effectively capture and discern subtle patterns within the motor imagery data is reflected in higher sensitivity values across diverse subjects. Conversely, in the evaluation of specificity, which gauges a classifier’s ability to correctly identify negative instances, Support Vector Machine (SVM) and RF exhibit notable prowess. Particularly, SVM stands out for its ability to minimize false positives, showcasing strong specificity across various subjects. RF, while excelling in sensitivity, also proves to be adept at maintaining specificity, making it a versatile choice for balancing the classification performance on both positive and negative instances.

### 3.2. A Comparative Analysis of Feature Selections

After feature extraction by using a self-attention deep learning model, the behavior of feature selection techniques that were used in this method is really important for our study. In this paper, we conducted a comprehensive evaluation of eight feature selection methods applied to MI classification tasks using the BCI Competition III dataset IV-a. After the selected feature extraction technique self-attention, eight feature selection techniques were employed on five subjects (aa, al, av, aw, ay) followed by the feature selection technique of Recursive Feature Elimination (RFE), Correlation feature selection (Cor), Linear Regression (LR), Neighborhood Component Analysis (NCA), Relief Feature Selection (ReliefF), Mutual information (MIFs), Independent Component Analysis (ICA), and Principal Component Analysis (PCA). The classification performance was assessed using a Support Vector Machine (SVM) as a best-performing classifier, and the results were analyzed to identify the most effective feature selection methods.

**Figure 8:**
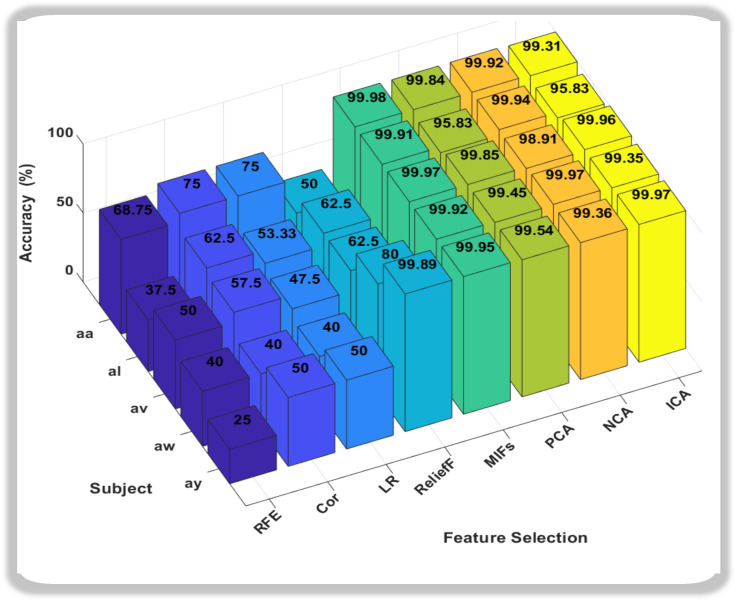
Performance parameters of Motor Imaginary dataset IV-a.

The results demonstrate that ReliefF, MIFs, ICA, and PCA consistently yield high classification accuracies across all subjects as shown in Figure 8. These methods exhibit superior performance compared to RFE, Cor, LR, and NCA. Notably, mutual information consistently achieves near-perfect accuracy, indicating its robustness in extracting relevant features for SVM classification in MI tasks. The observed trends suggest that the information captured by ReliefF, MIFs, ICA, and PCA is particularly discriminative for our chosen methodology.

The effectiveness of these methods may stem from their ability to uncover essential patterns and reduce dimensionality, leading to improved classification performance, as demonstrated in Table 1.

In the feature selection phase of our experiments, Recursive Feature Elimination (RFE) was applied following self-attention-based feature extraction. However, certain subjects, such as al, aw, and ay, exhibited lower classification accuracies, with values dropping below 50%. This reduction in accuracy is primarily due to the poor separation between the clusters of classes 1 and 2 in the feature space, which limits the effectiveness of RFE in identifying the most discriminative features.

RFE operates by recursively eliminating less important features based on the performance of a predictive model. However, the method is heavily reliant on the quality and separability of the feature set. In cases where class boundaries are ambiguous, or when features are highly redundant, RFE may fail to select the optimal subset of features, leading to suboptimal classification performance. Additionally, RFE can be sensitive to noise and computationally expensive, further impacting its performance in datasets with overlapping or poorly separated classes. These limitations highlight the need for more sophisticated or complementary feature selection methods when working with complex datasets.

**Figure 9:**
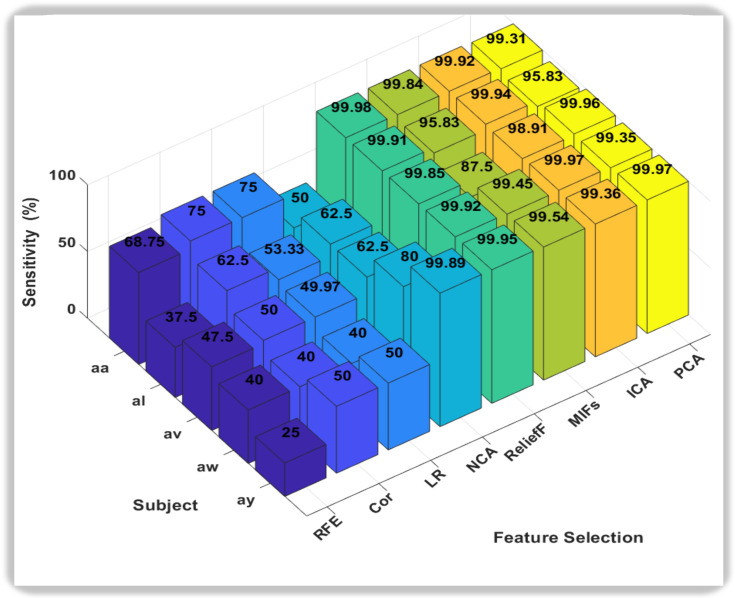
Specificity of MI dataset-Iva using SVM classifier.

**Figure 10:**
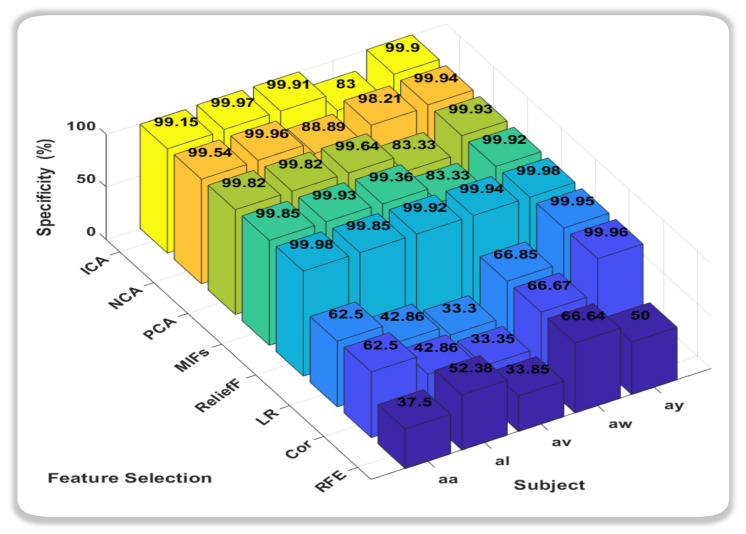
Specificity of MI task dataset Iva using SVM classifier.

The ratio of true positives to the sum of true positives and false negatives is used to know the value of sensitivity, also known as the true positive rate, while specificity, often known as the true negative rate, calculates the model’s ability to properly detect negative events. It is determined by dividing the number of true negatives by the total number of true negatives and false positives. In this paper, the 3D bar graph depicting sensitivity and specificity for each feature selection method across subjects reveals insightful patterns as shown in Figure 9 and Figure 10. ReliefF, Mutual Information, Independent Component Analysis (ICA), and Principal Component Analysis (PCA) have also emerged as standout performers in achieving a balance between sensitivity and specificity. These methods consistently exhibit high accuracy in identifying both positive and negative instances, making them well-suited for MI tasks. The robust performance of these feature selection techniques suggests their potential for enhancing the reliability of BCI systems, contributing to improved overall classification outcomes.

### 3.3. Comparison between Number of Selected Features

In our previous experiments conducted on the Motor Imagery (MI) dataset, Independent Component Analysis (ICA), Principal Component Analysis (PCA), Mutual Information Feature Selection (MIFs), and Relief Feature Selection (ReliefF) emerged as the most effective techniques for feature selection in the context of our MI task. Building upon these successful outcomes, we intend to further refine our feature set by selecting one feature from each trial and fifteen features from each trial.

In Table 2, where one top feature is selected from each trial of our MI task after feature extraction, distinct patterns emerge across the feature selection methods (MIFs, ReliefF, PCA, ICA) and machine learning models (SVM, LDA, KNN, RF, XGBoost). Notably, ReliefF consistently yields high accuracy across models, ranging from approximately 97.11% to 99.01%.

MIFs exhibit varied performance, with SVM achieving notably high accuracy values (up to 99.78%), while PCA consistently performs well, particularly with RF and XGBoost reaching accuracy values as high as 99.89%. ICA demonstrates consistently high performance across all models, with accuracy values ranging from 93.17% to 99.98%.

In Table 3, where the top 15 features are selected from each trial, there is a discernible improvement in accuracy for most feature selection methods and models. ReliefF, in particular, shows a notable boost in accuracy, ranging from approximately 97.11% to a perfect accuracy of 100% across all subjects.

MIFs, when selecting the top 15 features, result in a significant accuracy improvement, especially for SVM, achieving perfect accuracy (100%) in the case of subject “ay” and good results in other subjects. PCA maintains its strong performance, with high accuracy values across all models, including perfect accuracy for RF (100%) for all subjects.

ICA continues to exhibit high accuracy, with values ranging from 90.38% to a perfect 100%. Overall, selecting the top 15 features generally leads to improved accuracy compared to choosing only one top feature from each trial.

The results provide a deep observation into the effectiveness of different feature selection techniques and the impact of the number of features selected on the performance and accuracy of machine learning models in the Motor Imagery (MI) task.

In this paper, the feature selection method Mutual Information (MIFs) has consistently emerged as the most effective feature selection method for our research paper. After using various machine learning models, including SVM, LDA, KNN, RF, and XGBoost, MIFs consistently demonstrated the best performance in terms of accuracy for our MI data. The results consistently show high accuracy values, ranging from 92.31% to a perfect 100%, depending on the specific subjects and scenarios, such as selecting one top feature or the top 15 features from each trial of our MI data. This robust and steady performance underscores the importance of MIFs in optimizing feature sets for enhancing the predictive capabilities of the machine learning models that we used in our study. The findings reinforce the prominence of Mutual Information Feature Selection as a powerful technique in the domain of Motor Imagery analysis, providing a reliable and effective approach for feature selection in our experimental context.

**Figure 11:**
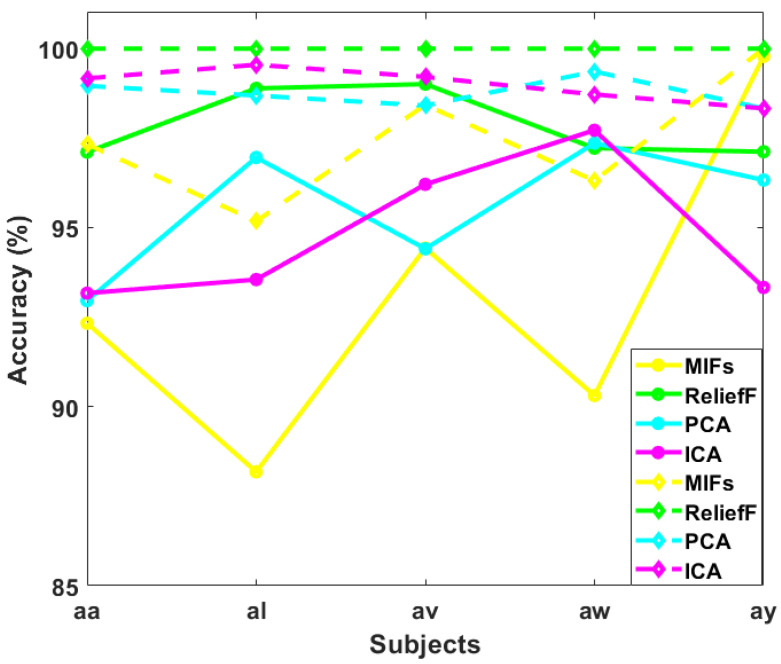
A graphical comparison between top one selected features and top 15 selected features.

In Figure 11, we present a graphical comparison illustrating the impact of selecting the top 1 feature and the top 15 features from each trial using various feature selection methods—MIFs, ReliefF, PCA, and ICA. The line plot distinctly represents the accuracies achieved by each feature selection method for both scenarios. The top 1 selected feature from each trial is depicted with solid lines, while the top 15 selected features are represented by dotted lines. The color scheme enhances the visual distinction, with MIFs depicted in yellow, ReliefF in green, PCA in cyan, and ICA in magenta. Notably, the graph demonstrates a consistent trend across all feature selection methods. The accuracies obtained with the top 15 selected features consistently outperform those achieved with only the top 1 selected feature from each trial. This observation aligns with the expectation that a richer set of features, as represented by the top 15 features, provides a more comprehensive and informative input to the machine learning models.

The dotted lines, indicating the top 15 features, consistently surpass the solid lines corresponding to the top 1 feature, underscoring the significance of selecting a more extensive set of features for enhanced model performance. This visual representation reinforces the efficacy of MIFs, ReliefF, PCA, and ICA in capturing and selecting features that contribute significantly to the task at hand. The distinct color-coded lines facilitate an easy comparison of the impact of different feature selection methods on model accuracy, providing valuable insights into the performance gains achieved by selecting a larger feature set in the context of our Motor Imagery task.

### 3.4. Correlation Matrix Analysis for Redundancy and Independence of Selected Features

**Figure 12:**
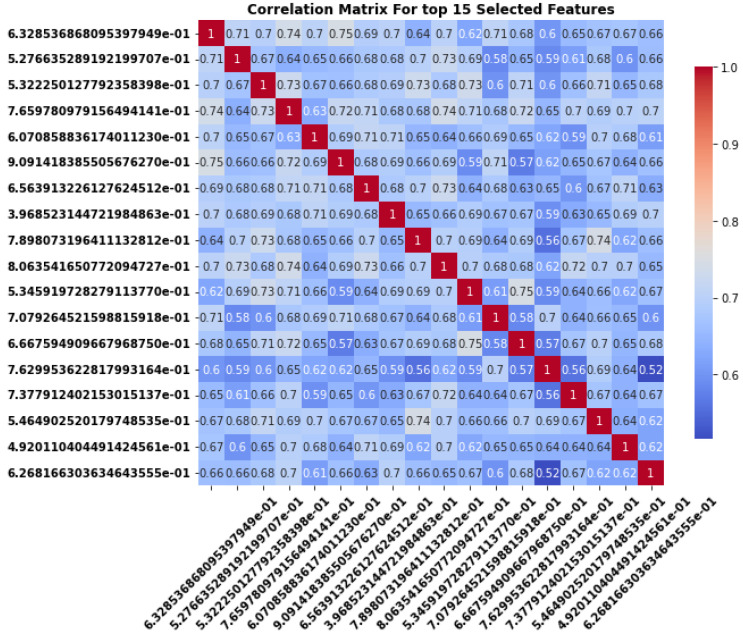
Correlation Matrix for top 15 selected features.

**Figure 13:**
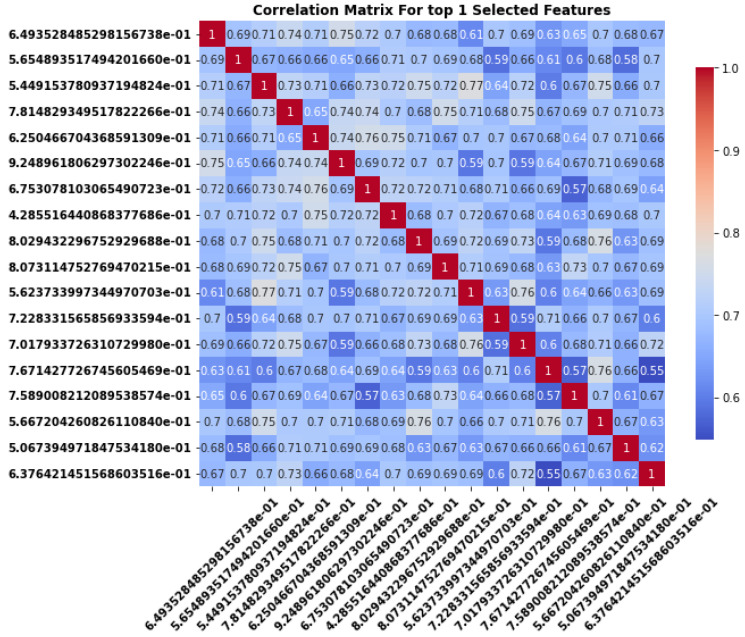
Correlation Matrix for top 1 selected features.

The correlation matrix shown in the Figure 12 and Figure 13 represents the pairwise relationships between the top 15 and top 1 selected features. Each cell in the matrix indicates the strength and direction of the linear relationship between two features, with values ranging from −1 to 1. A value of 1, indicated by the red diagonal, represents a perfect positive correlation where a feature is correlated with itself. Positive correlations (in red shades) imply that as one feature increases, so does the other, while negative correlations (in blue shades) suggest an inverse relationship where an increase in one feature corresponds to a decrease in the other.

In the case of the top 15 selected features many of the off-diagonal values show moderate to high positive correlations (ranging between 0.6 and 0.8), suggesting that several features are closely related to one another and may provide redundant information. For example, there are several clusters of correlations around 0.7 to 0.8, indicating that these feature pairs are strongly positively correlated. A few features, like those in the bottom-right portion of the matrix, show slightly lower correlations, suggesting more independent contributions to the dataset as shown in Figure 12.

In the case of the top 1 selected features, most of the off-diagonal values show moderate to strong positive correlations, with many values clustering between 0.6 and 0.9 as shown in Figure 13. This indicates that several of the selected features tend to increase or decrease together, suggesting that they may be capturing similar patterns in the dataset. For example, features in the top-left and bottom-right sections of the matrix show correlations close to 0.7 or higher, implying that these feature pairs are likely redundant.

Moreover, while some features exhibit moderate correlations, providing more diverse information, there are no significant negative correlations in the matrix. This suggests that the dataset lacks pairs of features that consistently behave in opposing directions. The absence of both strong negative correlations and near-zero correlations further highlights the potential redundancy among the selected features. When multiple features are highly correlated, as seen here, it may introduce issues like multicollinearity, especially in predictive modeling. Multicollinearity can distort the interpretation of models, particularly in linear regression, and may lead to overfitting where the model becomes too reliant on a small set of redundant features.

### 3.5. Five-Fold Validation

In our comprehensive analysis of feature selection methods for our MI task, we employed a rigorous five-fold validation strategy to evaluate the performance of four distinct techniques, Mutual Information Feature Selection (MIFS), Relief Feature Selection (ReliefF), Independent Component Analysis (ICA), and Principal Component Analysis (PCA). The first thing we conducted was to identify the most effective feature selection method that could enhance the accuracy of our MI task classification. Remarkably, each of these methods revealed promising results, showing the effectiveness of their respective approaches. Upon conducting the five-fold validation experiments, it became evident that the top-performing feature selection method varied across trials. The individual trials revealed that Mutual Information (MIFS), ReliefF, ICA, and PCA each had instances where they outperformed the others as the best feature selection method for each trial. This variability underscores the complexity of MI data and the importance of employing diverse feature selection techniques to adapt to different characteristics present in the data.

**Figure 14:**
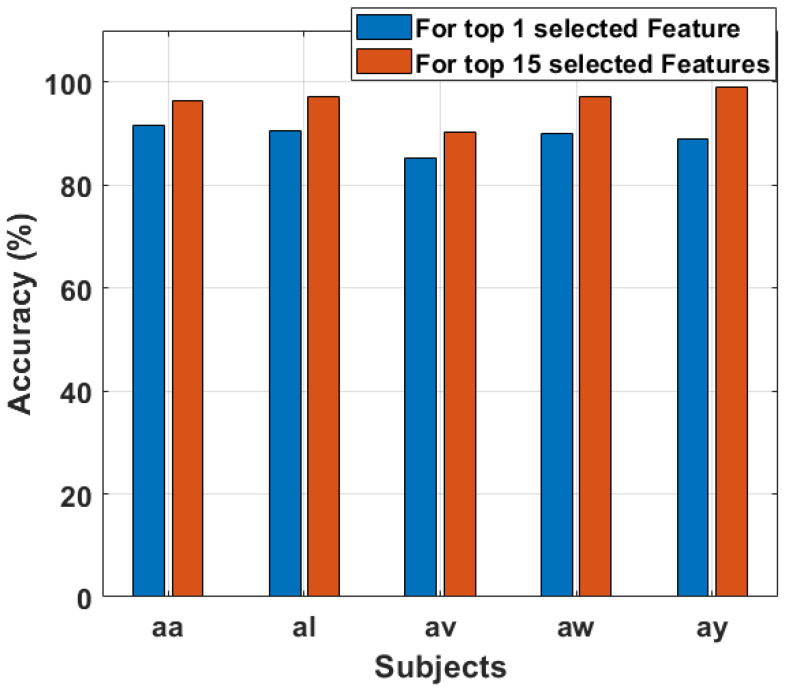
Comparison of Accuracy (%) of both scenarios by using MIFs as feature selection and SVM as classifier.

Surprisingly, not only did we observe strong accuracy results with our top-performing feature selection method, but a deeper exploration into the top 15 features selected by each method also revealed compelling performance. The inclusion of additional features beyond the single best feature showcased the robustness of the selected features and their collective impact on classification accuracy. This observation is particularly noteworthy as it suggests that a more comprehensive feature set, encompassing the top 15 selected features, consistently contributed to favorable outcomes in our five-fold validation experiments. Table 4 provides a detailed overview of the accuracies obtained through five-fold validation for both the top one best-selected feature and the top 15 best-selected features for each feature selection method. The tabulated results allow for a nuanced comparison between the performances of these two feature selection strategies. Figure 14 complements the tabular data by offering a visual representation of the accuracy trends, facilitating a quick and intuitive understanding of the comparative performances. The findings presented in Table 4 and Figure 14 collectively suggest that while the top one best-selected feature is crucial and often yields commendable results, the broader selection of the top 15 features consistently delivers competitive accuracies. This insight underscores the importance of exploring not only the best individual features but also the relation among a selected set of features in our MI task.

Overall, our in-depth analysis of feature selection methods for MI task classification has revealed a dynamic landscape where different trials may benefit from distinct feature selection techniques. The robustness of the top 15 selected features highlights the potential for a more inclusive approach to feature selection, leveraging the collective strength of a broader feature set. These results contribute valuable insights to the field of EEG signal processing, offering researchers and professional advisors of the field of BCI to select appropriate feature selection methods based on the specific characteristics of the MI data.

### 3.6. Effect of Denoised and Noised MI Data

Multi-scale Principal Component Analysis (MSPCA) has proven to be particularly effective for denoising EEG signals compared to other traditional methods. While conventional denoising techniques like wavelet thresholding or basic filtering methods focus on removing noise, they often risk distorting the underlying EEG signal or removing important low-amplitude components. MSPCA, however, leverages both the multi-scale nature of wavelets and the dimensionality reduction capabilities of PCA to efficiently separate noise from a signal without significantly altering the relevant features of the EEG data.

By decomposing the signal into different scales, MSPCA isolates noise present at various frequencies and removes it while preserving the underlying structure of the EEG signal. This makes it especially suited for handling the complex, non-stationary nature of EEG data, where both low-frequency brain activity and high-frequency noise coexist. In our study, the MSPCA-denoised signals demonstrated substantial improvement in signal quality, as evidenced by a notable increase in the signal-to-noise ratio (SNR) and a reduction in root mean square error (RMSE) compared to the raw signals.

Additionally, when applied to the classification pipeline, MSPCA-denoised signals consistently yielded higher accuracy across all classifiers, outperforming results obtained from other denoising techniques. The improvement in classification accuracy underscores the effectiveness of MSPCA in preserving the essential features of the EEG signal that contribute to model performance. Thus, MSPCA not only excels in noise reduction but also enhances the overall accuracy of EEG-based classification tasks, making it a superior method for EEG signal denoising compared to more conventional approaches.

In this paper, we performed a comprehensive analysis of Motor Imagery (MI) data, by comparing the performance between denoised and non-denoised datasets using the Multi-Scale Principal Component Analysis (MSPCA) method for denoising. Our primary goal was to assess the impact of denoising on feature selection and classification accuracy. The results that we obtained from the experiment shed light on the effectiveness of the MSPCA denoising technique in improving the overall performance of the MI classification task.

First, we began by selecting only the single best feature from each trial of our MI data and observed notable improvements in accuracy when using denoised MI data. The average accuracies expressed in percentages, 92.33%, 88.18%, 94.13%, 90.31%, and 99.87%, were for subjects aa, al, av, aw, and ay, respectively. Contrarily, when we used non-denoised data, the accuracies dropped noticeably to 71.12%, 64.59%, 85.16%, 80.56%, and 85.76% for each subject. This visible difference underscores the effectiveness of the MSPCA denoising method in enhancing the discriminatory power of selected features and subsequently improving the accuracy of the MI classification task that we used in this paper. Next, we extended our investigation to the scenario of selecting the top 15 best features from each trial. The results revealed an even more pronounced advantage for denoised data. When employing denoised MI data, the accuracies reached 97.33%, 95.18%, 98.43%, 96.31%, and 100% for subjects aa, al, av, aw, and ay, respectively. In contrast, without denoising, the corresponding accuracies were notably lower at 78.21%, 80.36%, 88.76%, 82.76%, and 89.79%.

This substantial improvement reaffirms the MSPCA denoising technique’s ability to extract and retain relevant information from MI signals, allowing the classifier to make more accurate predictions. Central to our experimental setup was the utilization of Support Vector Machines (SVM) as the classifier and Mutual Information (MI) as the feature selection technique. This combination proved effective in harnessing the denoised information, and the relationship between denoising and sophisticated classification methodologies. The results not only validate the robustness of SVM but also emphasize the crucial role of feature selection in optimizing classification accuracy.

**Figure 15:**
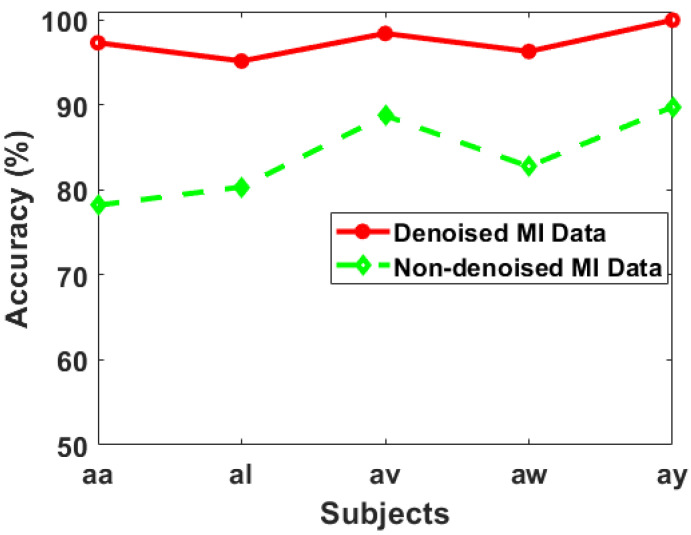
A graphical visualization of Denoised and Non-denoised data.

In Figure 15, a compelling graphical comparison unfolds, illustrating the impact of denoising on Motor Imagery (MI) data accuracy. The solid red line represents the accuracy trend derived from denoised MI data, while the green dotted line mirrors the accuracy of non-denoised data. This visual representation immediately communicates the profound effect of the denoising process on the classification performance of the MI task. The solid red line exhibits a notably higher and smoother trajectory, reflecting the accuracy achieved with denoised MI data and the red line consistently maintains a superior position compared to its non-denoised counterpart. This result implies that the denoising method, likely the Multi-Scale Principal Component Analysis (MSPCA) as mentioned earlier, effectively mitigates the adverse impact of noise, leading to enhanced feature discrimination and, consequently, improved accuracy.

Contrastingly, the green dotted line, representing the accuracy of non-denoised MI data and the visual disparity between the two lines underscores the significance of denoising techniques in the preprocessing phase of MI data analysis. Figure 15 vividly illustrates the performance gap between denoised and non-denoised MI data. The solid red line serves as a testament to the effectiveness of denoising in elevating the accuracy of the classification task. This comparison not only substantiates the importance of preprocessing methods in enhancing the quality of MI data but also underscores the potential of denoising techniques to contribute significantly to the reliability of Motor Imagery-based applications. The visual clarity provided by this graphical representation emphasizes the practical impact of denoising on the robustness and accuracy of MI data analysis, offering valuable insights for researchers and practitioners in the field of neurotechnology.

Our application of MSPCA as a denoising technique reflects the practical integration of established methods to enhance the extraction of meaningful features from MI data, mitigating the adverse effects of noise and enhancing the discriminative power of selected features. This finding underscores the importance of preprocessing techniques, such as denoising, in preparing raw MI data for effective utilization in machine learning tasks. Moreover, the substantial increase in accuracy when selecting the top 15 features further highlights the advantage of considering a broader set of features, made possible by our denoising approach. Our experiment showcases the transformative impact of MSPCA-based denoising on MI data, resulting in significantly improved classification accuracies. The findings underscore the potential of this approach to contribute to advancements in neurotechnology, neurorehabilitation, and Brain–Computer Interfaces, where accurate and reliable MI classification is paramount.

### 3.7. Deep Learning Classification

In this study, we utilized five advanced deep learning models—RNN, GRU, WebNet, CatBoost, and RestNet—to evaluate the performance of AtSiftNet after processing our data through Mutual Information feature selection. This method allowed us to identify the most relevant features, enhancing the classifiers’ ability to generalize and improve classification accuracy. The results, presented in both Table 5 and Table 6, illustrate the accuracy of each model with both the top one and top 15 selected features.

The results demonstrate a marked improvement in overall accuracy when transitioning from the top one selected features to the top 15 selected features across all models. For instance, the average accuracy for RNN increased from 73.638% to 78.67%, while GRU similarly rose from 84.43% to 88.58%.

CatBoost consistently outperformed the other models, achieving an average accuracy of 94.86% with the top one features and further increasing to 98.27% with the top 15 features. RestNet also exhibited robust performance, with average accuracies of 93.37% for the top one features and 98.126% for the top 15 features. Notably, RNN, while showing improvement, remained the lowest-performing model in both tables, suggesting a need for further optimization of its architecture.

The results further reveal that the deep learning methods employed in this study—RNN, GRU, WebNet, CatBoost, and RestNet—differ in their capacity to leverage the selected features effectively. For example, GRU and WebNet demonstrated substantial gains, with GRU’s accuracy improving from 84.43% to 88.58% and WebNet’s from 88.52% to 93.288%. These findings highlight the effectiveness of Mutual Information in identifying relevant features that enhance classification accuracy, particularly for the more advanced models like CatBoost and RestNet, which consistently deliver superior results in classification tasks.

### 3.8. Algorithmic Complexity

**Figure 16:**
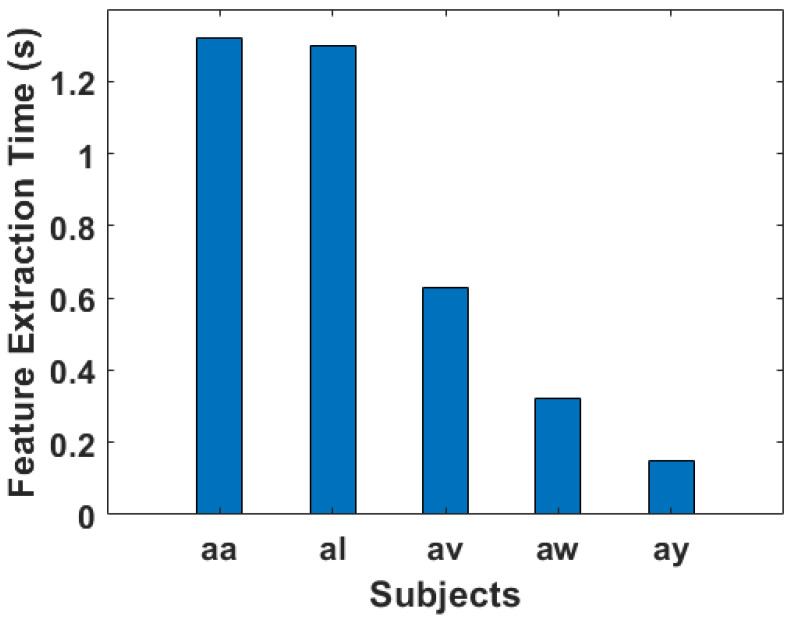
Feature Extraction time of each subject.

**Figure 17:**
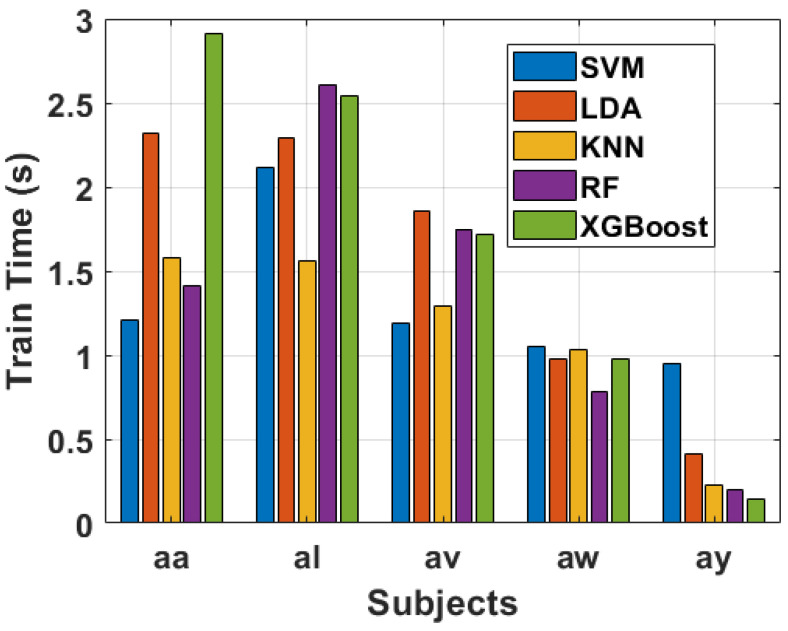
Using all classifier training time of each subject.

**Figure 18:**
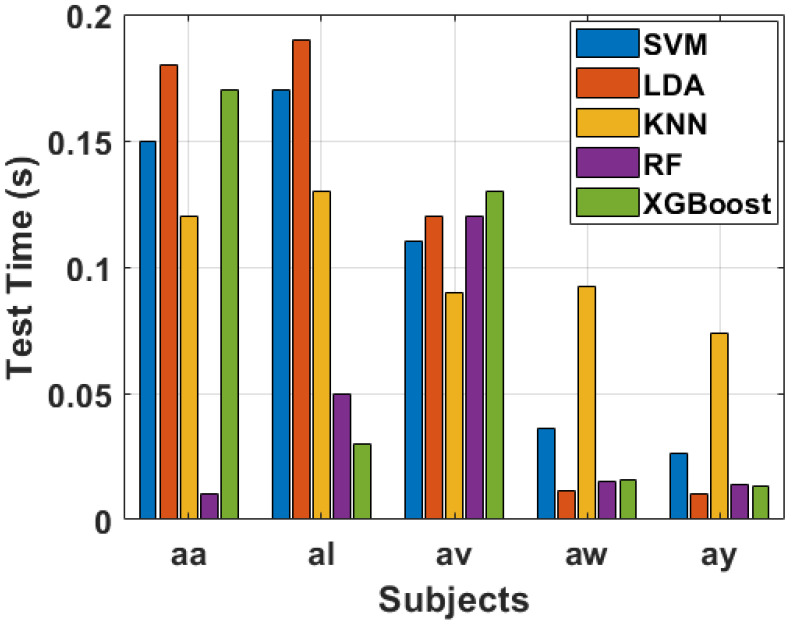
Using all classifier Testing Time of each subject.

The computational efficiency of the proposed method is assessed through various performance metrics, as depicted in Figure 16, Figure 17 and Figure 18. The feature extraction time for each subject, denoted as “aa”, “al”, “av”, “aw”, and “ay”, is presented in Figure 16. Notably, subject “aa” exhibits the longest feature extraction time, totaling 1.32 s, followed closely by subject “al” with 1.30 s. In contrast, subjects “av”, “aw”, and “ay” demonstrate significantly shorter feature extraction times of 0.63, 0.32, and 0.15 s, respectively. This variance can be attributed to the differing number of trials conducted by each subject, resulting in variations in the number of features extracted.

Moving on to the classification stage, Figure 17 illustrates the training times for Support Vector Machine (SVM), Linear Discriminant Analysis (LDA), k-Nearest Neighbors (KNN), Random Forest (RF), and XGBoost. The highest training time across all classifiers is observed for subject “al” with SVM, totaling 2.12 s. Meanwhile, subjects “av”, “aw”, and “ay” exhibit relatively lower training times, showcasing the computational effectiveness of the proposed method in our paper. The training times for LDA, KNN, RF, and XGBoost also vary across subjects, with the algorithms demonstrating distinct computational demands based on the underlying data characteristics.

In Figure 18, the testing times for each subject using the same set of classifiers are presented in a bar chart. In the case of subject “aa”, it displays the longest testing time across all classifiers, this subject with SVM yielding a testing time of 0.15 s. Conversely, subjects “aw” and “ay” reveal notably shorter testing times, emphasizing the efficiency of the proposed method in real-time clinical applications of MI data.

To provide a comprehensive overview of the computational performance, and how much time our proposed algorithm takes, Figure 16, Figure 17 and Figure 18 include bar charts for feature extraction time, training time, as well as testing time. These visual representations in bar charts offer a clear comparison of the computational demands imposed by different subjects and classifiers. The results underscore the practicality and efficiency of the suggested method, especially when compared to other feature extraction techniques. Thus, the novel proposed technique in our study demonstrates promising computational efficiency in the context of feature extraction, training, as well as testing across multiple subjects. The ability to handle varying trial sizes and feature sets makes it a viable candidate for real-time clinical applications. The thorough analysis presented in Figure 16, Figure 17 and Figure 18 provides valuable insights into the algorithm’s performance, aiding researchers and practitioners in understanding its strengths and potential areas for improvement.

### 3.9. ROC Curves and AUC Values

The performance of the classifier and feature selection technique was commonly evaluated by the Receiver Operating Characteristic (ROC) curve and Area Under the Curve (AUC) values. Where ROC is the graphical representation of the positive rate (sensitivity) and false positive rate (specificity) trade-off between them among the different threshold values, while AUC is a scalar value under the curve that shows the overall performance of any classifier or feature selection method; values range between 0 and 1.

The ROC curves and corresponding AUC values provide a comprehensive evaluation of various feature selection techniques applied in our experiment. Each technique, namely Linear Regression, ReliefF, Mutual Information, Correlation, Recursive Feature Elimination (RFE), Principal Component Analysis (PCA), Neighborhood Component Analysis (NCA), and Independent Component Analysis (ICA), exhibits distinctive performance characteristics.

Linear Regression and Correlation with AUC values of 0.59 demonstrate modest discriminative power. These methods may struggle to capture the complex relationships within the data, resulting in suboptimal predictive performance. On the contrary, ReliefF achieves an AUC of 1.00, indicating perfect discriminatory ability. ReliefF excels in identifying relevant features by considering feature interactions and is particularly robust in scenarios with intricate relationships.

Mutual Information, PCA, and ICA also exhibit outstanding performance, boasting AUC values of 1.00. These techniques excel in capturing non-linear dependencies and intricate patterns within the data, making them robust choices for feature selection in scenarios where feature interactions are crucial. The superior performance of Mutual Information, PCA, and ICA underscores their ability to discern relevant features effectively, contributing to the overall predictive power of the model.

RFE, with an AUC of 0.44, reveals comparatively lower discriminative ability. This technique might struggle in scenarios with high dimensionality or intricate feature dependencies, impacting its performance in accurately selecting relevant features. NCA, with an AUC of 0.86, demonstrates good discriminative power but falls short of the perfect scores achieved by ReliefF, Mutual Information, PCA, and ICA.

**Figure 19:**
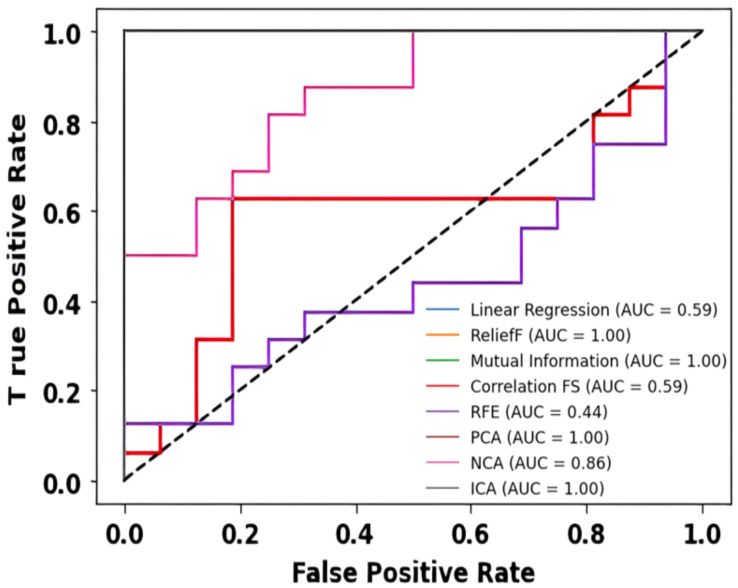
ROC curves and AUC values of feature selection technique.

In the visual representation provided in Figure 19, the ROC curves further illustrate the trade-offs between sensitivity and specificity for each feature selection technique. Techniques with higher AUC values exhibit curves that approach the upper-left corner, indicating superior performance. The stark differences in AUC values highlight the varying efficacy of each method in capturing the underlying structure of the data. Thus, the choice of feature selection technique significantly influences the model’s predictive performance.

ReliefF, Mutual Information, PCA, and ICA emerge as top performers, excelling in capturing relevant features and intricate relationships within the data. These findings underscore the importance of thoughtful feature selection in enhancing the overall robustness and accuracy of predictive models in the context of our experiment.

**Figure 20:**
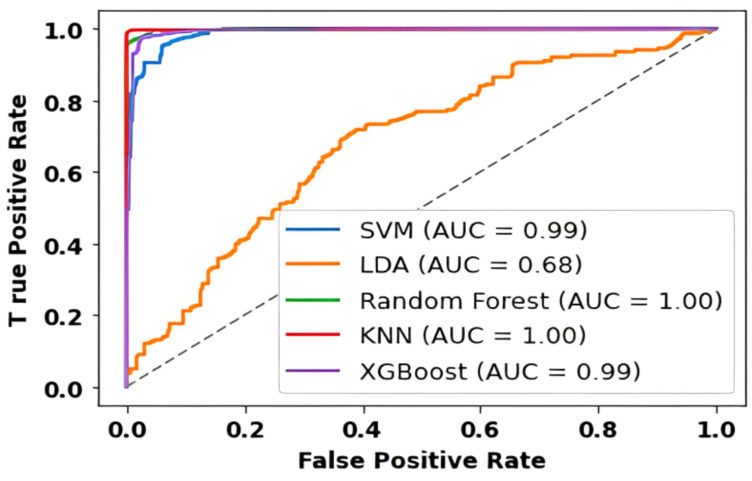
ROC graphs by using MIFs as a feature selection technique.

The AUC values obtained from our experiment provide valuable insights into the performance of different classifiers across subjects and feature selection strategies in the context of Motor Imagery (MI) tasks. We conducted two sets of experiments, one utilizing the top-best single selected feature from each trial and another employing the top 15 selected features from each MI task trial, all based on Mutual Information (MIFs). The ROC graph of the 15 best-selected features using mutual information as a feature selection technique is shown in Figure 20. In the case of using the top-best single selected feature, SVM consistently demonstrated exceptional performance across all subjects, achieving perfect AUC values of 1. This suggests that SVM excels in accurately discriminating between classes when utilizing the most informative feature from each trial. This robust performance is noteworthy and underscores SVM’s capability to effectively leverage individual features for precise classification in MI tasks.

LDA also demonstrated strong performance, with AUC values ranging from 0.77 to 0.99 across subjects. While slightly below the perfect score achieved by SVM, LDA’s ability to capture discriminative information from individual features is evident. KNN and XGBoost, too, demonstrated consistent high AUC values, indicating their effectiveness in leveraging the top-best single selected feature for accurate classification. RF, although performing well, exhibits slightly lower AUC values compared to SVM, LDA, KNN, and XGBoost.

When considering the top 15 selected features from each MI task trial, the classifiers’ performance dynamics shift. SVM maintains strong discriminatory power with AUC values ranging from 0.95 to 1, reaffirming its resilience when dealing with a larger feature set. Interestingly, LDA’s performance fluctuates, demonstrating a wider range of AUC values across subjects. While still effective, it suggests that LDA may be more sensitive to the composition of the feature set. KNN and RF continue to perform admirably with perfect AUC scores for most subjects. XGBoost exhibits strong performance but with slight variability, showcasing its sensitivity to feature selection.

**Figure 21:**
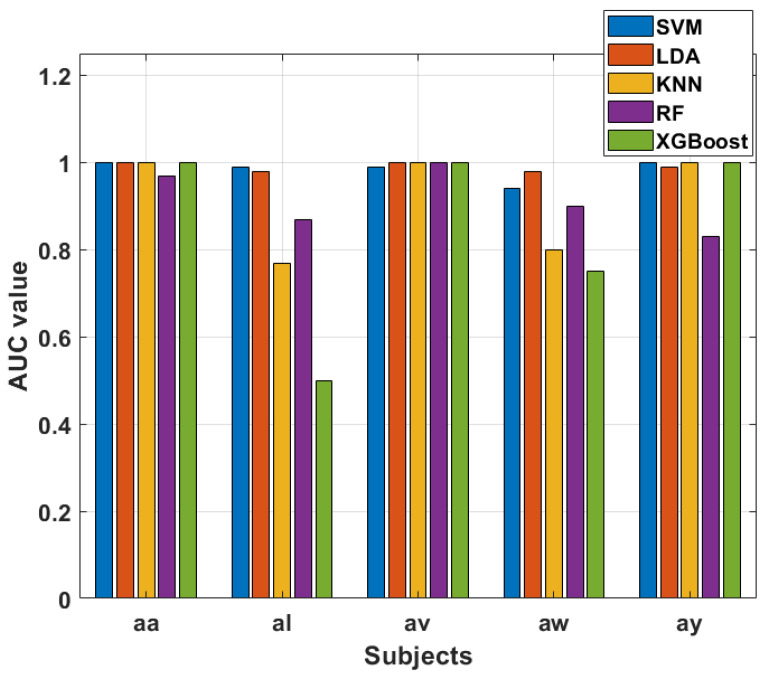
Comparison of AUC values for top one selected features from each trial.

**Figure 22:**
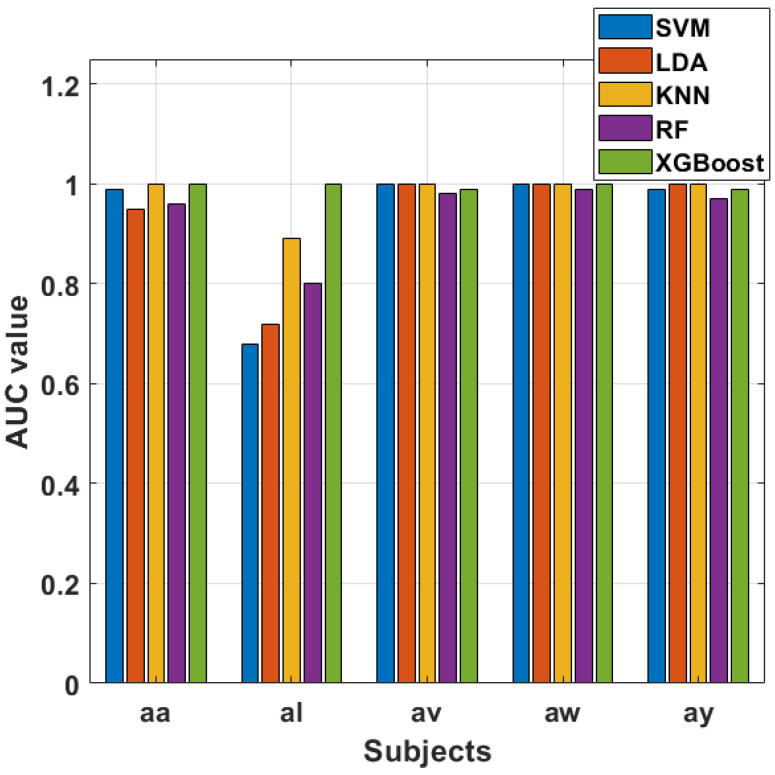
Comparison of AUC values for top 15 selected features from each trial.

The contrast between the two scenarios (top-best single feature vs. top 15 features) emphasizes the impact of feature selection on classification outcomes. Figure 21 and Figure 22 visually represent the AUC values, providing a clear comparison between the classifiers and the two feature selection scenarios. These visualizations highlight the strengths and potential limitations of each classifier and underscore the importance of thoughtful feature selection in optimizing classification outcomes for MI tasks. Hence, our experiments show the performance of classifiers across different subjects and feature selection strategies in MI tasks. SVM consistently stands out as a robust performer, particularly when leveraging individual features, while other classifiers like LDA, KNN, RF, and XGBoost demonstrate varying degrees of effectiveness depending on the feature selection strategy employed.

### 3.10. Comparison with Other Methods

In this comprehensive study, the focus was on evaluating the accuracy of a proposed method across multiple subjects, denoted as aa, al, av, aw, and ay. The results reveal impressive performance metrics, with individual subject accuracies ranging from 99.68% to a perfect 100%. Specifically, subjects aw and ay achieved flawless accuracy rates of 100%, underlining the robustness of the proposed self-attention feature extraction method. Here, subject “aa” shows outstanding accuracy, leading all other state-of-the-art methods with a remarkable 99.96%. Subject “av” also demonstrates outstanding performance, with a noteworthy accuracy of 99.98%, leading all other state-of-the-art methods in the case of the MI task.

The overall average (Avg) accuracy for all subjects was calculated at an impressive 99.924%, illustrating the consistency and solidity of the proposed method, as demonstrated in Table 7. A notable aspect of the study is the achievement of a 100% classification accuracy for subjects aw and ay, showcasing the efficacy of the proposed method in handling specific subjects with great precision. The attention to detail extends to the comparison of the proposed method with others, revealing that the method attains the highest average classification accuracy of 99.55%, leading all other studies in the case of BCI competition III dataset IVa.

One exceptional aspect of the proposed method is its consistency, as evidenced by the low standard deviation (Std) value of 0.52 in our case. This low variability indicates the reliability and stability of the results across all subjects of the MI dataset. Such consistency is crucial in applications where precision and repeatability are paramount.

Thus, the study provides compelling evidence for the effectiveness of the proposed self-attention feature extraction method in achieving high classification accuracies across a diverse set of subjects. The meticulous comparisons with state-of-the-art methods, along with the detailed analysis of individual subject performances, collectively contribute to positioning the proposed method as a standout approach in the field, demonstrating not only superior accuracy but also remarkable consistency in its results.

### 3.11. Future Curiosity

While our present research has yielded promising results and demonstrated the efficacy of the proposed self-attention feature extraction method in binary classification tasks across subjects aa, al, av, aw, and ay, it is essential to acknowledge the existing limitations and chart a course for future directions. As we reflect on the advantages of our approach, it becomes apparent that the scope of our investigation has been confined to specific EEG domains, namely the subjects mentioned, without exploring broader applications in domains such as dementia, schizophrenia, alcoholism, and stroke disorders.

One significant avenue for future exploration lies in extending the application of our method to diverse EEG domains. By diversifying the scope of our research, we can unlock the potential of the proposed self-attention feature extraction method to contribute to the understanding and diagnosis of neurological conditions beyond the current binary classification framework. Investigating the applicability of our method in domains characterized by distinct EEG patterns and complexities could open new avenues for diagnostic and therapeutic interventions. For instance, exploring its efficacy in identifying patterns associated with dementia or schizophrenia could pave the way for novel insights and advancements in these critical areas of neuroscience.

Another critical dimension for future research involves transcending the binary classification paradigm. While our method has excelled in distinguishing between two classes, the transition to multiclass classification scenarios holds great promise [36,37]. Expanding the model’s capabilities to accommodate multiple classes would enhance its versatility and applicability across a broader spectrum of EEG-based applications. This evolution could involve adapting the self-attention mechanism to effectively discern and classify EEG patterns associated with different cognitive states or neurological conditions, contributing to a more nuanced and comprehensive understanding of brain activity.

Furthermore, our future research endeavors aim to explore subject-independent experiments. Currently, our methodology has been tailored to the characteristics of specific subjects (aa, al, av, aw, and ay). However, extending the applicability of our method to a subject-independent framework represents a critical step toward enhancing its generalizability and real-world utility. Subject-independent experiments would involve training the model on data from one set of subjects and evaluating its performance on completely unseen subjects. This approach simulates a more realistic scenario, where the model’s effectiveness is tested across a diverse population, reinforcing its potential for practical applications in varied clinical or research settings. As we move forward, collaboration and interdisciplinary engagement become imperative. Collaborating with experts in neurology, psychiatry, and related fields can enrich our understanding of the nuanced EEG patterns associated with different neurological conditions. Such collaborations can guide the refinement and adaptation of our method to address the unique challenges posed by each domain [38].

Hence, our future research endeavors are poised to address the limitations of our current study and elevate the impact of the proposed self-attention feature extraction method. By venturing into unexplored EEG domains, transitioning to multiclass classification, and embracing subject-independent experiments, we aspire to contribute to the broader landscape of neuroscience research. Through these concerted efforts, our aim is to not only advance the scientific understanding of brain activity but also pave the way for practical applications that hold the potential to transform diagnostics and treatments in the realm of neurological and psychiatric disorders.

## 4. Conclusions

In this paper, we introduce a novel AtSiftNet method for classifying motor imagery (MI) data in Brain–Computer Interface (BCI) systems, tested on the BCI Competition III dataset IV-a. Our approach begins with careful organization and preprocessing of the dataset, including denoising using Multi-Set Principal Component Analysis (MSPCA) to improve the signal-to-noise ratio. The self-attention technique is then applied, proving effective in extracting distinct features from MI data. We compared the performance of the top single and top 15 feature selection methods, with the latter consistently outperforming the former. Our results show improved accuracy with denoised EEG signals, validating our preprocessing steps. Benchmarking against other state-of-the-art methods, our approach demonstrates superior average classification accuracy and stability across diverse datasets. This work advances MI data classification in BCIs, underscoring the effectiveness of our method and its potential for real-world applications in neurotechnology.

## Figures and Tables

**Table 1:** Classification Accuracy for different Feature selections in percentage (%).

	aa	al	Av	aw	ay	Avg. Accuracy
RFE	68.75	37.5	50	40	25	44.25
Correlation	75	62.5	57.5	40	50	53.16
LR	75	53.33	47.5	40	50	57
NCA	50	62.5	62.5	80	99.89	70.97
ReliefF	99.98	99.91	99.97	99.92	99.95	98.88
MIFs	99.84	95.83	99.85	99.45	99.54	98.90
ICA	99.92	99.94	98.91	99.97	99.36	99.62
PCA	99.31	95.83	99.96	99.35	99.97	99.94

**Table 2:** Accuracy in (%) of top one best feature from each trial of MI task.

		aa	al	av	aw	ay
MIFs	SVM	92.33	88.18	94.43	90.31	99.787
	LDA	88.79	64.96	82.13	70.44	94.67
	KNN	93.375	94.80	95.01	96.07	98.52
	RF	91.7	93.76	94.61	92.51	96.334
	XGBoost	95.08	94.32	92.42	90.94	89.667
ReliefF	SVM	97.11	98.89	99.01	97.22	97.12
	LDA	68.11	70.11	44.03	48.95	47.07
	KNN	40.86	64.889	51.57	48.23	46.12
	RF	61.44	62.45	44.031	46.22	45.214
	XGBoost	63.66	64.67	44.42	47.52	47.212
PCA	SVM	92.96	96.96	94.41	97.36	96.33
	LDA	91.38	93.24	90.06	94.15	93.67
	KNN	54.7	55.77	35.31	72.04	77.31
	RF	97.98	98.39	97.18	99.87	96.32
	XGBoost	96.12	94.85	96.52	98.89	94.334
ICA	SVM	93.17	93.55	96.21	97.72	93.33
	LDA	94.33	91.92	93.52	95.15	94.67
	KNN	95.60	90.38	91.31	88.38	93.66
	RF	97.125	99.98	96.98	99.98	97.58
	XGBoost	96.79	97.85	95.94	99.23	92.667

**Table 3:** Accuracy (%) of the top 15 best features from each trial of MI data.

		aa	al	av	aw	ay
MIFs	SVM	97.33	95.18	98.43	96.31	100
	LDA	94.79	66.96	84.13	72.44	96.67
	KNN	99.375	98.80	97.01	98.07	100
	RF	97.7	97.76	97.61	95.51	98.334
	XGBoost	97.08	97.32	96.42	92.94	91.667
ReliefF	SVM	99.96	99.68	99.98	100	100
	LDA	71.11	71.11	46.03	49.95	49.07
	KNN	42.86	68.889	53.57	50	49.12
	RF	64.44	64.45	46.031	49.22	49.214
	XGBoost	66.66	66.67	46.42	49.52	49.212
PCA	SVM	98.96	98.96	98.41	99.36	98.33
	LDA	94.38	95.24	92.06	96.15	96.67
	KNN	57.7	58.77	37.31	73.04	79.31
	RF	100	100	100	100	100
	XGBoost	100	99.85	100	100	98.334
ICA	SVM	99.17	99.55	99.21	98.72	98.33
	LDA	98.33	97.92	96.52	96.15	96.67
	KNN	99.60	95.38	94.31	90.38	96.66
	RF	100	100	99.98	100	100
	XGBoost	99.79	99.85	99.94	100	96.667

**Table 4:** Mean Accuracy in (%) of five-fold Validation.

	Feature Selection Method	Classifiers	aa	al	av	aw	ay
**Top one selected feature**	MIFs	SVM	91.5 ± 2.34	90.56 ± 0.88	85.29 ± 4.40	90.0 ± 4.9	89.0 ± 1.0
		RF	93.99 ± 2.80	98.65 ± 0.78	94.04 ± 3.72	90.91 ± 11.50	94.12 ± 3.02
	ReliefF	SVM	95.32 ± 3.12	99.97 ± 0.52	99.52 ± 0.14	98.99 ± 1.1	95.21 ± 3.25
		RF	94.12 ± 1.27	98.89 ± 0.03	99.42 ± 0.03	98.52 ± 1.03	94.28 ± 2.29
	ICA	SVM	93.52 ± 0.25	99.11 ± 0.21	96.47 ± 4.71	98.0 ± 3.64	95.23 ± 1.59
		RF	93.21 ± 2.13	99.46 ± 0.23	99.82 ± 0.16	98.18 ± 3.64	85.0 ± 0.20
	PCA	SVM	90.32 ± 0.52	98.22 ± 0.59	94.12 ± 3.72	94.36 ± 7.37	95.00 ± 9.01
		RF	93.12 ± 1.03	99.56 ± 0.89	99.21 ± 0.16	99.32 ± 0.01	90.0 ± 9.05
**Top 15 selected features**	MIFs	SVM	96.23 ± 0.82	97.40 ± 0.27	90.30 ± 0.78	97.06 ± 1.55	99.0 ± 1.33
		RF	95.12 ± 2.05	98.98 ± 0.64	97.68 ± 0.64	95.51 ± 0.99	98.33 ± 2.11
	ReliefF	SVM	98.21 ± 0.32	99.99 ± 0.01	99.98 ± 0.1	99.51 ± 0.09	99.96 ± 0.03
		RF	96.52 ± 3.01	99.01 ± 0.12	99.89 ± 0.1	99.97 ± 0.01	99.97 ± 0.03
	ICA	SVM	97.12 ± 0.12	99.40 ± 0.27	99.40 ± 0.27	99.85 ± 0.63	99.0 ± 1.33
		RF	96.89 ± 1.21	99.99 ± 0.03	99.99 ± 0.06	99.99 ± 0.01	99.99 ± 0.01
	PCA	SVM	98.32 ± 0.52	99.08 ± 0.52	98.59 ± 0.63	99.08 ± 0.52	98.67 ± 1.94
		RF	99.89 ± 0.32	99.98 ± 0.12	99.98 ± 0.02	99.98 ± 0.01	99.99 ± 0.01

**Table 5:** Accuracy in (%) for Top one Selected Features.

	aa	al	Av	aw	ay	Avg. Accuracy
RNN	72.14	68.32	75.16	82.38	70.19	73.638
GRU	86.57	84.18	85.17	85.91	80.32	84.43
WebNet	90.16	85.17	94.36	90.18	82.76	88.52
CatBoost	95.76	92.62	96.18	97.81	97.97	94.86
RestNet	90.73	92.18	88.13	97.64	98.17	93.37

**Table 6:** Accuracy in (%) for Top 15 Selected Features.

	aa	al	Av	aw	ay	Avg. Accuracy
RNN	78.19	74.702	80.18	85	75.29	78.67
GRU	89.78	88.10	92.16	87.67	85.22	88.58
WebNet	95.10	90.77	98.79	94.16	87.52	93.288
CatBoost	99.78	97.47	99.12	98.24	96.76	98.27
RestNet	96.54	98.48	95.74	99.89	99.98	98.126

**Table 7:** A brief comparison of our proposed method with other existing methods.

Researchers	Method Proposed	Accuracy of Classifiers in (%)						
		**aa**	**al**	**av**	**aw**	**ay**	**Avg**	**Std**
AtSiftNet method	Self-Attention + ReliefFfor top 15 bestselected featuresfrom each trial	99.96	99.68	99.98	100	100	99.924	0.52
Shiam et al. [27]	CSP	86.36	97.51	76.20	95.81	95.38	90.25	8.98
Kabir et al. [28]	SRCFS + LDA	88.03	97.98	74.17	94.76	95.31	90.05	9.60
Jian et al. [29]	CSP-R-MF	81.43	92.41	70.00	83.57	85.00	82.48	8.15
Sadiq et al. [25]	EWT + IA2 + LS-SVM	94.5	91.7	97.2	95.6	97	95.2	2.3
	MEWT + JIA + MLP	95	95	95	100	100	97	2.7
Singh et al. [30]	R-MDRM	81.25	100	76.53	87.05	91.26	87.21	7.8
Mahamune et al. [31]	stdWC	84.5	98.1	72.8	95.1	92.5	88.8	9.1
Tiwari et al. [32]	DCRCC	93.6	79.2	94.6	85.54	84.94	87.58	6.46
Park et al. [33]	CSCC	89.29	98.21	73.47	92.86	89.29	88.62	9.22
Miao et al. [34]	CTFSP	86.07	98.57	52.14	96.07	92.14	85.00	8.96
Maher et al. [35]	PSVM-BCI	99.20	98.70	99.41	99.39	99.50	99.24	7.6

## Data Availability

The original data presented in the study are openly available in Fraunhofer FIRST, Intelligent Data Analysis Group (https://www.bbci.de/competition/iii/desc_IVa.html).
